# Comparing the relative and absolute effect of erenumab: is a 50% response enough? Results from the ESTEEMen study

**DOI:** 10.1186/s10194-022-01408-w

**Published:** 2022-03-19

**Authors:** Raffaele Ornello, Carlo Baraldi, Simona Guerzoni, Giorgio Lambru, Anna P. Andreou, Bianca Raffaelli, Astrid Gendolla, Piero Barbanti, Cinzia Aurilia, Gabriella Egeo, Sabina Cevoli, Valentina Favoni, Fabrizio Vernieri, Claudia Altamura, Antonio Russo, Marcello Silvestro, Elisabetta Dalla Valle, Andrea Mancioli, Angelo Ranieri, Gennaro Alfieri, Nina Latysheva, Elena Filatova, Jamie Talbot, Shuli Cheng, Dagny Holle, Armin Scheffler, Tomáš Nežádal, Dana Čtrnáctá, Jitka Šípková, Zuzana Matoušová, Alfonsina Casalena, Maurizio Maddestra, Stefano Viola, Giannapia Affaitati, Maria Adele Giamberardino, Francesca Pistoia, Uwe Reuter, Simona Sacco

**Affiliations:** 1 Neuroscience Section, Department of Applied Clinical Sciences and Biotechnology, University of L’Aquila, Via Vetoio 1, L’Aquila, Italy; 2 PhD school in neurosciences; Department of biomedical, metabolic and neural sciences, University of Modena and Reggio Emilia, Modena, Italy; 3 Medical toxicology - Headache and Drug Abuse Research Center; Department of biomedical, metabolic and neural sciences, University of Modena and Reggio Emilia, Modena, Italy; 4 The Headache Service, Guy’s and St Thomas’ NHS Foundation Trust, Westminster Bridge Road, London, SE1 7EH UK; 5 Department of Neurology, Charité - Universitätsmedizin Berlin, Berlin, Germany; 6 Private Practice, Essen, Germany; 7 Headache and Pain Unit, IRCCS San Raffaele, Rome, Italy; 8 San Raffaele University, Rome, Italy; 9 IRCCS Istituto delle Scienze Neurologiche di Bologna, Bologna, Italy; 10 Headache and Neurosonology Unit, Fondazione Policlinico Universitario Campus Bio-Medico, Rome, Italy; 11 Headache Center, Department of Medical, Surgical, Neurological, Metabolic, and Aging Sciences, University of Campania “Luigi Vanvitelli”, Naples, Italy; 12 Headache Centre, Ospedale S. Antonio Abate, ASST Valle Olona, Gallarate, Italy; 13 Headache Centre, Division of Neurology and Stroke Unit, “A. Cardarelli” Hospital, Naples, Italy; 14 Sechenov First Moscow State Medical University (Sechenov University), Moscow, Russian Federation; 15 Southwest Neurology Audit and Research group (SoNAR), Department of Neurology, Derriford Hospital, Plymouth, PL6 8DH UK; 16 Department of Neurology, Alfred Health, Melbourne, VIC Australia; 17 Department of Neurology, West German Headache Center, University hospital Essen, Essen, Germany; 18 Military University Hospital Prague, Department of Neurology, 1st Faculty of Medicine Charles University, Prague, Czech Republic; 19 Motol University Hospital Prague, Department of Neurology, 2nd Faculty of Medicine Charles University, Prague, Czech Republic; 20 Department of Neurology, “G. Mazzini” Hospital, Teramo, Italy; 21 Department of Neurology, “F. Renzetti” Hospital, Lanciano, Chieti, Italy; 22 Department of Neurology, “S. Pio da Pietrelcina” Hospital, Vasto, Chieti, Italy; 23 Headache Center, Geriatrics Clinic, Department of Medicine and Science of Aging and Center for Advanced Studies and Technology (CAST), G. D’Annunzio University, Chieti, Italy; 24 Universitätsmedizin Greifswald, Greifswald, Germany

**Keywords:** Erenumab, Treatment efficacy, Migraine frequency, Real-world

## Abstract

**Background:**

Monoclonal antibodies acting on the calcitonin gene-related peptide (CGRP) or its receptor have changed migraine preventive treatment. Those treatments have led to reconsidering the outcomes of migraine prevention. Available data mostly considered benefits in terms of relative efficacy (percent or absolute decrease in monthly migraine days [MMDs] or headache days compared with baseline). However, not enough attention has been paid to residual MMDs and/or migraine-related disability in treated patients. In the present study, we aimed at comparing the relative and absolute efficacy of erenumab.

**Methods:**

ESTEEMen was a collaborative project among 16 European headache centers which already performed real-life data collections on patients treated with erenumab for at least 12 weeks. For the present study, we performed a subgroup analysis on patients with complete data on MMDs at baseline and at weeks 9-12 of treatment. Starting from efficacy thresholds proposed by previous literature, we classified patients into 0-29%, 30-49%, 50-74%, and ≥75% responders according to MMD decrease from baseline to weeks 9-12 of treatment. For each response category, we reported the median MMDs and Headache Impact test-6 (HIT-6) scores at baseline and at weeks 9-12. We categorized the number of residual MMDs at weeks 9-12 as follows: 0-3, 4-7, 8-14, ≥15. We classified HIT-6 score into four categories: ≤49, 50-55, 56-59, and ≥60. To keep in line with the original scope of the ESTEEMen study, calculations were performed in men and women.

**Results:**

Out of 1215 patients, at weeks 9-12, 381 (31.4%) had a 0-29% response, 186 (15.3%) a 30-49% response, 396 (32.6%) a 50-74% response, and 252 (20.7%) a ≥75% response; 246 patients (20.2%) had 0-3 residual MMDs, 443 (36.5%) had 4-7 MMDs, 299 (24.6%) had 8-14 MMDs, and 227 (18.7%) had ≥15 MMDs. Among patients with 50-74% response, 246 (62.1%) had 4-7 and 94 (23.7%) 8-14 residual MMDs, while among patients with ≥75% response 187 (74.2%) had 0-3 and 65 (25.8%) had 4-7 residual MMDs.

**Conclusions:**

The present study shows that even patients with good relative response to erenumab may have a clinically non-negligible residual migraine burden. Relative measures of efficacy cannot be enough to thoroughly consider the efficacy of migraine prevention.

**Supplementary Information:**

The online version contains supplementary material available at 10.1186/s10194-022-01408-w.

## Background

Monoclonal antibodies acting on the calcitonin gene-related peptide (CGRP) or its receptor have opened a new era of migraine treatment, as they are the first class of preventive drugs targeting a migraine-specific mechanism [1, 2]. Erenumab, a monoclonal antibody targeting the CGRP receptor, reported a high efficacy in randomized clinical trials [3–5] which was similar or even higher in real-world studies [6–15].

The availability of highly effective and specific migraine preventive treatments might lead to reconsider the efficacy outcomes. The threshold for a clinically meaningful response to migraine preventive treatments, including erenumab, is commonly set as a relative measure, i.e. a ≥50% decrease in the number of monthly migraine days (MMDs) compared with baseline [3–5]. In patients with chronic migraine, even a 30% decrease in MMDs has been considered relevant by some authors [16]. However, patients with a clinically meaningful response may still have a relevant absolute number of residual MMDs. In that view, the international definition of chronic migraine [17], as well as the most recent definition of resistant or refractory migraine [18], are based on an absolute number of MMDs or headache days; therefore, reporting absolute outcomes and analyzing their difference from relative ones can be important in clinical practice and in research settings.

The aim of the present study is to evaluate both the relative and absolute efficacy of erenumab in a large population of patients from real-world practice, in order to assess the difference between the two approaches and provide clinically relevant insights.

## Methods

### Study population

The present study is a subgroup analysis of the Efficacy and Safety of Treatment with ErEnumab in Men (ESTEEMen) study, a pooled patient-level analysis of real-world data referring to treatment with erenumab, whose details were previously published [19]. The ESTEEMen study was the result of a collaboration among 16 headache centers having already performed real-life studies on erenumab treatment for migraine prevention, with a minimum follow-up of 12 weeks. The analysis was approved by the Internal Review Board of the University of L’Aquila with protocol number 07/2021; ethical approval to pool data from each center was obtained if needed. All included patients were followed-up for 12 weeks, irrespective of treatment discontinuation.

### Data collection

Baseline was considered as the four weeks preceding the start of erenumab treatment, while outcomes were assessed at weeks 9-12 of treatment compared with baseline. For centers that reported a 30-day or longer baseline, values were normalized to a 4-week (i.e., 28-day) period to ensure comparability.

MMDs, monthly days of use of acute medication and triptans were collected in all centers by using headache diaries. Headache Impact Test-6 (HIT-6) scores were also collected at baseline and at weeks 9-12. For the purposes of the present study, we used data on MMDs and HIT-6 scores.

We classified the categories of response as 0-29%, 30-49%, 50-74%, and ≥75%, according to the reduction in MMDs at weeks 9-12 compared with baseline. Patients with missing data on MMDs due to data collection issues were excluded from the analyses. Patients with available MMDs at baseline and stopping the treatment before 12 weeks because of inadequate response, adverse events, or loss to follow-up were included in the present analysis using a “last observation carried forward” approach.

### Statistical analysis

Descriptive statistics were reported as numbers and proportions and medians with interquartile ranges (IQRs), as appropriate. We assessed the proportion of 0-29%, 30-49%, 50-74%, and ≥75% responders in our sample. For each response category, we reported the median MMDs and HIT-6 scores at baseline and at weeks 9-12; median values at baseline were compared with those at weeks 9-12 by using Wilcoxon test. We also categorized the number of residual MMDs at weeks 9-12 as follows: 0-3, 4-7, 8-14, ≥15. Those categories follow important thresholds, as 4 MMDs identify a need for prevention, 8 MMDs can identify high frequency episodic migraine – or chronic migraine if coupled with ≥15 monthly headache days –, and ≥15 MMDs identify a definite condition of chronic migraine. We classified HIT-6 into four categories: ≤49, 50-55, 56-59, and ≥60 [20]. The distributions of MMD and HIT-6 categories at weeks 9-12 and at baseline were compared using the chi-square test. To keep in line with the scope of the ESTEEMen study, calculations were performed in men and women.

We used a convenience sample without any formal sample size calculation. Missing HIT-6 values were imputed using the median score value.

## Results

Out of 1410 patients included in the ESTEEMen dataset, 195 (13.8%) had missing data due to data collection issues; we included in the analyses 1175 (83.3%) patients with complete MMD data both at baseline and at weeks 9-12 and 40 (2.8%) patients with missing data due to early treatment stopping; 860 (70.8%) of those 1215 patients also had complete data on HIT-6 scores. The median age of patients was 49 years (IQR 41-56); 985 (81.1%) patients were women and 230 (18.9%) men; 936 patients (77.0%) had CM and 279 (23.0%) episodic migraine.

**Fig. 1:**
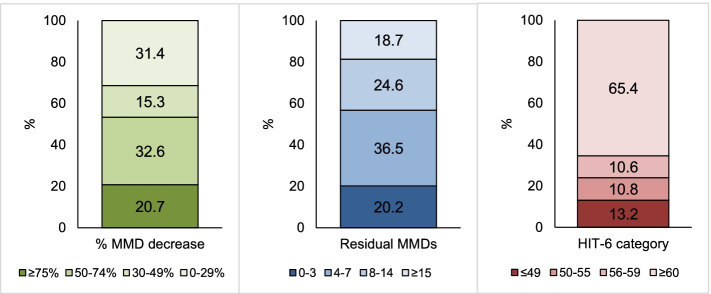
Relative (percent decrease in monthly migraine days) and absolute (residual monthly migraine days and Headache Impact Test-6 score) response to erenumab

**Fig. 2:**
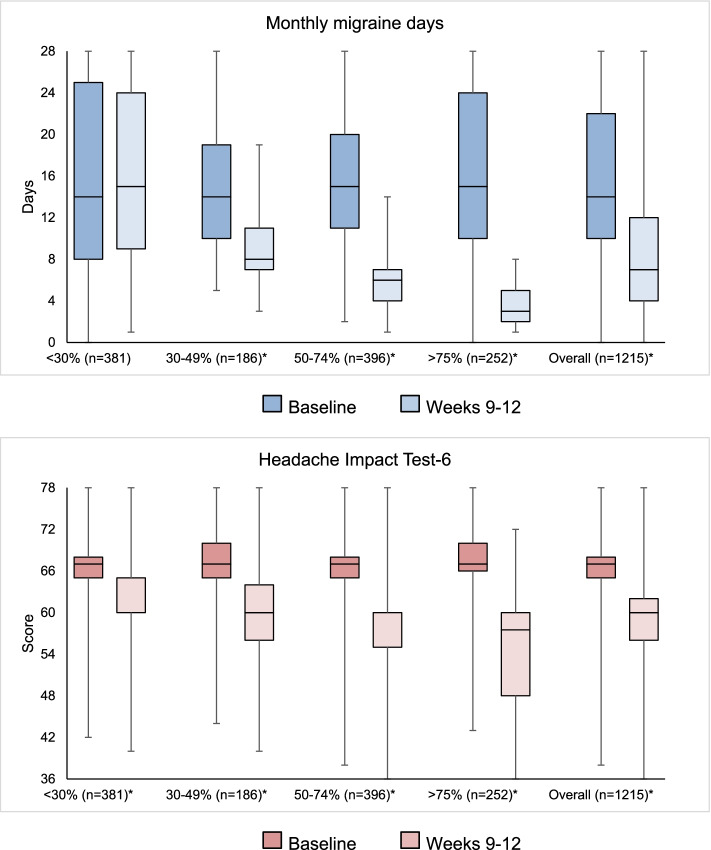
Box plots showing the change in monthly migraine days (above) and HIT-6 scores (below) from baseline to weeks 9-12 of treatment with erenumab

Figure 1 shows the distribution of response, residual MMD, and HIT-6 score categories at weeks 9-12. Median MMDs decreased from 14 (IQR 10-22) to 7 (IQR 4-12) in the overall group (*P*<0.001); MMD decrease was significant in each response group except from patients with 0-29% response (Fig. 2-A; Supplemental Table 1). Median HIT-6 score decreased from 67 (IQR 65-68) to 60 (IQR 56-62) in the overall group (*P*<0.001); HIT-6 decrease was significant in each response group (Fig. 2-B; Supplemental Table 2).

**Fig. 3:**
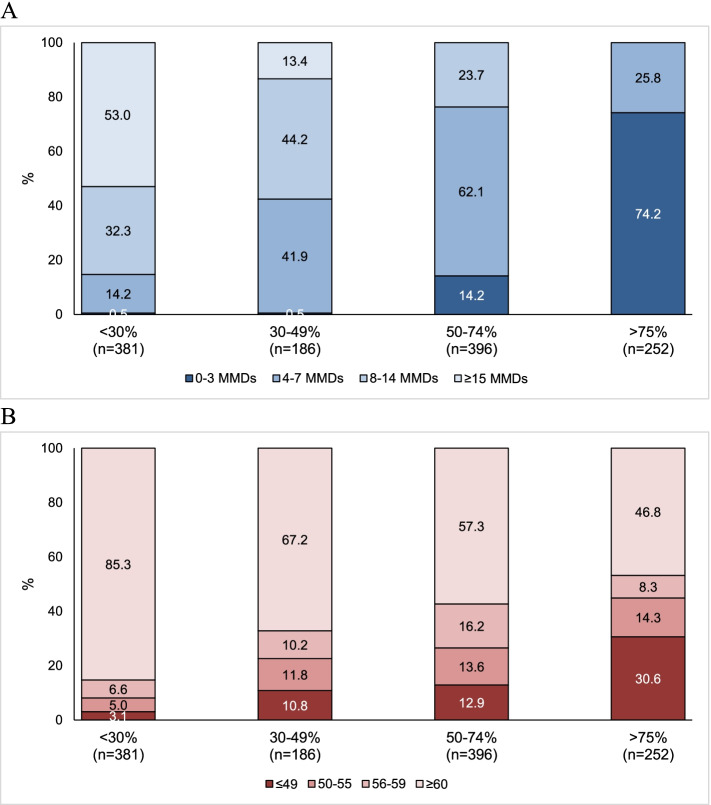
Residual monthly migraine days (**A**) and Headache Impact Test-6 score (**B**) at weeks 9-12 according to erenumab response categories

Figure 3 shows residual MMDs and HIT-6 scores at weeks 9-12 according to response categories. Among patients with ≥75% response rate, the residual migraine burden was low as all patients had less than 8 residual MMDs and 187 (74.2%) had 0-3 residual MMDs. Patients with 50-74% response rate showed a higher migraine burden as 94 (23.7%) had ≥8 MMDs and 246 (62.1%) 4-7 MMDs. Among patients with 30-49% response rate, only 79 (42.4%) had less than 8 residual MMDs. Referring to HIT-6 score categories, the proportions of patients with high migraine-related disability (score ≥60) were high in all response rate categories, ranging from 46.8% in patients with ≥75% response up to 85.3% in those with 0-29% response.

**Fig. 4:**
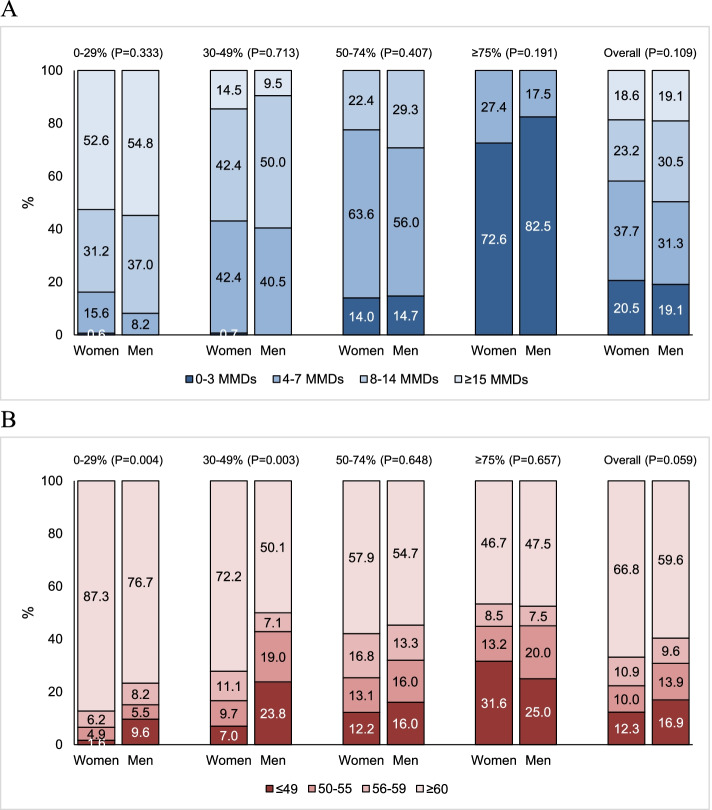
Residual monthly migraine days (**A**) and Headache Impact Test-6 score (**B**) at weeks 9-12 according to erenumab response categories in men and women

Figure 4 shows gender-specific data on residual MMDs and HIT-6 score categories at weeks 9-12 compared with baseline. Residual MMDs did not show gender differences, while HIT-6 distribution was less favorable in women than in men in the 0-29% (*P*=0.004) and in the 30-49% (*P*=0.003) response categories (Fig. 4).

## Discussion

The percent decrease in MMDs from baseline is an important clinical outcome in patients with migraine; however, it may lead to overlook an important migraine burden in some patients. Especially in CM patients, a high relative decrease in MMD may correspond to a still high number of residual MMDs that may cause impairment in quality of life and daily activities and could even require additional prevention. Our study aimed at reporting the differences between the relative and absolute efficacy of erenumab in a large real-life population. Relative efficacy was reported as the percent decrease in MMDs from baseline to weeks 9-12, while absolute efficacy was reported as the absolute decrease in MMDs and as the number of residual MMDs. Additionally, the residual migraine impact was reported as the HIT-6 score at weeks 9-12. A comparison between absolute and relative efficacy of erenumab was already performed in subgroup analyses of randomized controlled trials [21, 22]; in those previous analyses, patients with a 50% or 75% response had a substantially higher decrease in MMDs compared with those with lower relative efficacy. However, the previous analyses did not report the number of residual MMDs, which is important to consider the actual efficacy of migraine prevention as already been suggested by some authors [23]. Reporting the number of residual MMDs allowed to show that some patients with a 50-74% response might not have an optimal reduction of their migraine burden. In our study, most patients with ≥75% response had an optimal response with 0-3 residual MMDs, while 5 out of 6 patients with 50-74% response can be placed above the threshold of 4 residual MMDs per month; 62.0% met the criteria for high-frequency episodic and 23.9% for chronic migraine. It is important to note that the number of residual MMDs was substantial in patients with 30-49% response and close to that of patients with a 0-29% response. Hence, in our opinion, a 30% decrease in MMDs from baseline could not be considered an acceptable response to migraine prevention, even in patients with chronic migraine. Future basic and clinical research will likely identify novel targets to provide pain relief to those patients. While basic migraine research is advancing, it may be worthwhile to prolong the use of the available treatments, especially in those patients who have failed all the other preventatives, as a small benefit is still better than none. Monoclonal antibodies acting on the CGRP are particularly suitable for long-term treatments, given their favorable safety profile and acceptance by patients [24].

In addition to those considerations, we also found that migraine-related disability, measured by the HIT-6 score, behaved differently from migraine frequency. The score decreased in all subgroups of patients; however, it stayed relatively high even after an effective treatment with erenumab. About half of patients with a 50-74% or even ≥75% response had a substantial impact of migraine on daily life according to the score. This finding suggests that residual MMDs can be associated with a high migraine-related disability even after optimal pharmacological prevention.

Our findings should be put in the context of the real-world use of erenumab and of the other monoclonal antibodies acting on the CGRP pathway. Most of those patients fulfill the definition of resistant or even refractory migraine [18], which identifies patients with multiple preventive treatment failures. Those patients represent a population with a high burden of migraine-related disability [25]. Even if the advent of monoclonal antibodies acting on the CGRP pathway has revolutionized the therapeutic expectancies of those patients, their migraine burden may remain high [26]. Clinical practice suggests that combined treatments with oral drugs and/or non-pharmacological interventions could provide a further substantial benefit to patients with suboptimal treatment with monoclonal antibodies; however, there are no available data on the efficacy of add-ons to monoclonal antibodies, except from observational studies of combination with onabotulinumtoxinA [27–31]. An algorithm for the use of onabotulinumtoxinA for chronic migraine already proposed combination with oral agents in patients with partial response to the injective treatment [32]; the same can apply to erenumab and other monoclonal antibodies. Personalized treatment based on the residual burden of migraine could be important to optimize prevention and improve quality of life. Relative measures of drug efficacy could be useful to understand whether a treatment should be continued, while absolute measures could identify the need for further therapeutic efforts. Decreasing MMDs below 4 is unrealistic for most patients, and mostly those with CM; however, other cutoffs of absolute efficacy could be useful for a thorough patient evaluation.

The previous analysis of the ESTEEMen data did not show gender difference in the response to erenumab [19]. In this subgroup analysis, we showed that even the residual migraine burden was comparable in men and women. As noted in the previous analysis, this finding can be explained by the fact that patients treated with erenumab are a highly selected population of patients, in which gender differences might have been attenuated. The gender difference found in migraine-related disability in patients with 0-29% and 30-49% response was slight and probably irrelevant on a clinical point of view.

The main strength of the present study is its large population from specialized headache centers. Although limited to a single drug, our findings could be generalized to any monoclonal antibody acting on the CGRP pathway. Moreover, this is the first study assessing the residual migraine burden after a preventive treatment. However, the study also suffers from many limitations. The 12-week follow-up is short and migraine outcomes could vary over longer courses of treatment. The design of the study did not allow the collection of many variables, which could not allow to consider possible confounders. Results are generalizable to patients with CM, but not to those with episodic migraine as they represent a minority of patients treated with erenumab. We could not compare data about overall headache days, as the most complete data were available only for MMDs. Besides, HIT-6 values were not complete and were replaced by median values, which could have affected the results. In addition, HIT-6 assesses migraine-related disability in a different fashion as compared with other tools, such as the Migraine Impact and Disability Assessment Scale (MIDAS), which were not used in the present study. Lastly, our database did not allow to consider the effect of concomitant oral preventive treatments and of medical comorbidities in patients treated with erenumab.

## Conclusions

Our data show that there is a difference between the relative and absolute efficacy of migraine preventatives in patients with migraine. Overall, a higher relative efficacy was associated with better absolute outcomes; however, even in patients who can be considered as responders by relative response parameters, the residual migraine burden may be high. In our opinion, a single measure of relative efficacy such as percent decrease in MMDs, although easy to assess, cannot be enough. Thorough evaluation of patients with migraine considering residual burden and the therapeutic expectations is always to be considered. Further research is needed to address unmet needs in migraine prevention and the possible benefits of combination or treatment switch in selected patients.

## Supplementary Information

**Additional file 1.**

## Data Availability

The datasets used and/or analysed during the current study are available from the corresponding author on reasonable request.

## Abbreviations

CGRP: Calcitonin gene-related peptide
CM: Chronic migraine
ESTEEMen: Efficacy and Safety of Treatment with ErEnumab in Men
HIT-6: Headache Impact Test-6
IQR: Interquartile range
MMDs: Monthly migraine days
