# Supplementary material for: Comparing the relative and absolute effect of erenumab: is a 50% response enough? Results from the ESTEEMen study
Source: J Headache Pain. 2022 Mar 19;23(1):38. doi: 10.1186/s10194-022-01408-w (PMC8933935; doi:10.1186/s10194-022-01408-w)
Supplement: Supplementary file 1 — Additional file 1. [file 10194_2022_1408_MOESM1_ESM.docx]

**Supplemental Table 1**. Decrease in median monthly migraine days from baseline to weeks 9-12 according to response categories.

| **Category** | **Baseline** | **Weeks 9-12** | **P value** |
| --- | --- | --- | --- |
| 0-29% | 14 (9-26) | 15 (9-24) | 0.494 |
| 30-49% | 14 (10-19) | 8 (7-11) | <0.001 |
| 50-74% | 15 (11-20) | 6 (4-7) | <0.001 |
| ≥75% | 15 (10-24) | 2 (1-4) | <0.001 |
| Overall | 14 (10-22) | 7 (4-12) | <0.001 |

Data are expressed as medians and interquartile ranges.

**Supplemental Table 2**. Decrease in median Headache Impact Test-6 score from baseline to weeks 9-12 according to response categories.

| **Category** | **Baseline** | **Weeks 9-12** | **P value** |
| --- | --- | --- | --- |
| 0-29% | 67 (65-68) | 60 (60-65) | <0.001 |
| 30-49% | 67 (65-70) | 60 (56-64) | <0.001 |
| 50-74% | 67 (65-68) | 60 (55-60) | <0.001 |
| ≥75% | 67 (66-70) | 57.5 (48-60) | <0.001 |
| Overall | 67 (65-68) | 60 (56-62) | <0.001 |

Data are expressed as medians and interquartile ranges.
